# Highly Sensitive and Precise Analysis of SF_6_ Decomposition Component CO by Multi-comb Optical-feedback Cavity Enhanced Absorption Spectroscopy with a 2.3 μm Diode Laser

**DOI:** 10.1038/s41598-019-46190-z

**Published:** 2019-07-04

**Authors:** Fu Wan, Feng Zhou, Jin Hu, Pinyi Wang, Jianxin Wang, Weigen Chen, Chengzhi Zhu, Ye Liu

**Affiliations:** 1 State Key Laboratory of Power Transmission Equipment &System Security and New Technology, Chongqing University, Chongqing, 400044 P.R. China; 2 College of Materials Science and Engineering, Chongqing University, Chongqing, 400044 P.R. China; 3 State Grid Zhejiang Electric Power Company, LTD, Zhejiang, 310007 P.R. China

**Keywords:** Optical spectroscopy, Optical spectroscopy

## Abstract

A ppb-level CO sensor based on multi-comb optical-feedback cavity enhanced absorption spectroscopy with a 2.3 μm diode laser was developed for SF_6_ decomposition analysis in electric power system. The effective optical path reached to 4.5 km within 35 cm length cavity. Besides, through modulating the cavity length five times automatically, the spectral resolution was improved to 0.0015 cm^−1^ from 0.0071 cm^−1^. Targeting the *R*(6) line of CO first overtone band at 4285.01 cm^−1^, which is interference free from absorption spectra of SF_6_ mixtures (SF_6_, SO_2_, H_2_S, SO_2_F_2_, HF, CF_4_, CO_2_, COS, O_2_ and H_2_O), the minimum detection limit and detection precision under different gas pressures were performed. At optimum integration time of 30 s determined by Allan deviation analysis and gas pressure of 40 torr, the minimum detection limit and detection precision of CO were better than 18 ppb and 150 ppt, respectively.

## Introduction

Sulfur hexafluoride (SF_6_) is a kind of inert gas with stable chemical properties and has excellent insulating and arc-extinguishing abilities, which are widely used as an insulating gas in gas insulated electrical equipment of power system, such as gas insulated switchgear, transmission pipes and gas circuit breaker. When insulation faults occur in these gas insulated electrical equipment, such as overheating and partial discharge, the SF_6_ will decompose and react with C, H and O atom generated from organics or the impurities (such as O_2_, H_2_O) in SF_6_, to form various fault characteristic gases (such as CO, SO_2_, H_2_S, SO_2_F_2_, HF, CF_4_, CO_2_ and COS), which can be used as an indicator to identify fault types of gas insulated electrical equipment^1,2^.

The existing research indicates that CO is one of the key fault characteristic gases. CO can reflect fault of equipment overheating, under which, organic solid insulating materials such as polyester ethylene, insulating paper and insulating paint will slowly decompose and release CO gas. Besides, CO can also be used as a criterion to evaluate and predict the severity and developing trend of partial discharge fault, such discharge fault will provide energy to break molecular bonds of O_2_ to produce O atom, which will react with C atom from decomposition of organic solid insulating materials, then CO releases. Therefore, there is considerable interest in developing an online, cost-effective, highly sensitive and precise CO sensor in SF_6_, SO_2_, H_2_S, SO_2_F_2_, HF, CF_4_, CO_2_, COS, O_2_ and H_2_O for fault diagnosis of gas insulated electrical equipment^3–5^. The minimum detection limit of CO sensor should achieve 200 ppb according to the Institution of Engineering and Technology and China Power Industry standard^6^.

Recently, trace gas sensors based on tunable diode laser absorption spectroscopy (TDLAS) are widely used due to their high detection sensitivity and selectivity as well as fast response time, which permits online monitoring of target gas concentrations^7–10^. The basic principle of tunable diode laser absorption spectroscopy can be described briefly as: (a) The emission wavelength of diode laser, is tuned over the characteristic absorption lines of a target gas; (b) Then, the gas concentration can be determined through the reduction of the measured signal intensity caused by gas absorption^11^. In 2007, F. Wang *et al*.^12^ developed a TDLAS based sensor for CO detection in CO_2_ by used of a diode laser operating at 1.58 μm, which resulted in a minimum detection limit of 200 ppm. In 2009, X. Chao *et al*.^13^, used the TDLAS technique and a 2.3 μm distributed feedback (DFB) diode laser to monitor CO in N_2_, CO_2_ and H_2_O with a minimum detection limit of ~3 ppm. In 2017, J. Dang *et al*.^14^ combined TDLAS with techniques of wavelength modulation spectroscopy and multipass gas cell, and adopted a high-power, continuous wave (CW) DFB QCL emitting at 4.7 μm to detect CO in N_2_, a minimum detection limit of 26 ppb was achieved. What is more, R. Ghorbani *et al*.^15^ presented a compact sensor for CO isotopes in air and exhaled breath based on a 4 m circular multiple gas cell with an interband cascade laser operating at 4.69 µm, which resulted in a minimum detection limit of 9 ppb. Although a ppb-level CO sensor was easily achieved by combined the TDLAS technique with a mid-IR laser targeting the strongest fundamental ro-vibrational transitions of CO, these CO sensors were developed to operate in an air or exhaled gases environment and may not adapt to detect trace CO in various fault characteristic gas mixtures because the cross interference from absorption of SF_6_, SO_2_, H_2_S, SO_2_F_2_, HF, CF_4_, CO_2_, COS, O_2_ and H_2_O (SF_6_ mixtures) cannot be ignored. In 2018, R. Cui *et al*.^16^ had proven TDLAS CO sensor in mid-IR region is unsuitable for SF_6_ decomposition analysis since SF_6_ in high concentration levels (usually > 99.99%) has abundant absorption spectra in infrared area and will strongly interfere the fundamental ro-vibrational band of CO. Based on the test of pure SF_6_ (99.99%) by Fourier Transform Infrared (FTIR) spectrometer and simulation of SO_2_, H_2_S, SO_2_F_2_, HF, CF_4_, CO_2_, COS, O_2_ and H_2_O based on HITRAN database, R(6) transitions located at 4285.01 cm^−1^ in the first overtone band of CO was selected as the target detection absorption line, which is interference free from absorption spectra of SF_6_ mixtures. Combined with 2 *f* wavelength modulation spectroscopy and TDLAS technique, the minimum detection limit of CO sensor developed by R. Cui reached 1 ppm. However, this value is not meet the detection limit required by the standards^6^.

Optical feedback cavity enhanced technique not only can increase the coupling efficiency of the laser radiation into the gas cell but also can improve effective optical path length between laser and gas sample^17–19^. Combined with lasers in mid-IR or single mode laser diodes in near-IR region, the minimum detection limit of optical feedback cavity enhanced absorption spectroscopy can easily reach ppt or ppb, respectively. Besides, many researchers have been demonstrated that a compact, stable and robust spectrometer based on optical feedback cavity enhanced absorption spectroscopy is ideally suited for field measurements^20–23^.

In this paper, to our best knowledge, we first describe CO sensor system based on TDLAS and optical feedback V-shaped cavity enhanced technique with a CW diode laser at 2.3 μm for SF_6_ decomposition detection. A piezoactuator attached to the rear of an end mirror of V-shaped cavity is constructed to allow the cavity length to be carefully modulated and change the position of the comb of build-up modes, thus, more data points per frequency unit and a high lineshape precision are achieved. This optical sensor system realizes highly sensitive and precise analysis of SF_6_ decomposition component CO in SF_6_, SO_2_, H_2_S, SO_2_F_2_, HF, CF_4_, CO_2_, COS, O_2_ and H_2_O at the target absorption line of 4285.01 cm^−1^.

## Sensor Design

**Figure 1:**
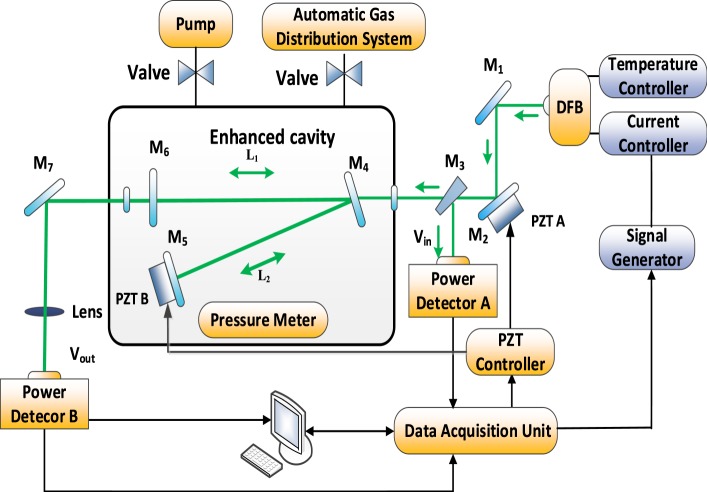
The schematic diagram of the multi-comb optical feedback V-shaped cavity enhanced absorption spectroscopy platform for CO measurement. The beam enters from the right. PZT A is used for satisfying the optical feedback phase. L_1_ = L_2_ = 35 cm (where L_1_ and L_2_ are the lengths of the two cavity arms), while PZT B is used for modulating the length of the cavity to get a high lineshape precision for better detection precision.

An experimental scheme of multi-comb optical feedback V-shaped cavity enhanced absorption spectroscopy is depicted in Fig. 1. In order to target the selected R(6) absorption line of CO at 4285.01 cm^−1^, a CW monomode DFB laser diode at 2334 nm from Nanosystems and Technologies GmbH is employed as the light source, which is regulated by a temperature controller (TCU151) and home-made current controller. At a typical temperature of 30 °C and 85 mA current, the diode laser provides 12 mW laser radiation with a temperature and current tuning coefficients of −0.412 cm^−1^/°C and −0.051 cm^−1^/mA, respectively.

A wedged ZnSe Mirror, M_3_, acting as a beam splitter is placed before the optical cavity to monitor the post-cavity signal, V_in_, for calibrating power loss resulted from gas absorption, using InGaAs detector A (Thorlabs, PDA 10D-EC). Another part of laser will transmit through an anti-reflection coated ZnSe window (Crystran) of sample chamber made of stainless steel and comes into a 30° V-shaped optical cavity consists of a planar folding mirror (at the center), M_4_, and two spherical concave mirrors (at the ends), M_5_ and M_6_ (radius of curvature 1 m), which are dielectrically coated CaF_2_ with a reflectivity of 99.99% on the front face (Lohnstar Optics). The laser will reflect back and forth multiple times in the V-shaped cavity, the arm length of which is 35 cm long, giving 70 cm unfolded cavity length. The spectral resolution is equal to 1/2 (*L*_1_ + *L*_2_) ≈ 0.0071 cm^−1^ without adjustment of cavity length. The V-shape has the same effect with a Faraday isolator which ensures that the direct back reflection from M_4_ does not return to the laser and causes wrong frequency locking, but it is more simple and take use of laser power when compared to isolator^17^. The diode laser beam decaying from M_6_ will be simultaneously monitored by InGaAs detector B, getting cavity output power, V_out_, to give the true value of gas absorption with the help of V_in_. A piezoactuator (PZT, Physik Instrumente, S-314.10) integrated into M_2_ mount provided precisely adjustment of the laser-to-cavity distance to equal to V-shaped cavity arm length, L_1_ + L_2_, which ensures phase matching that all cavity resonant frequencies, upon excitation, return to the laser with the same phase. When phase matching, a relatively large amount of radiation circulated in the cavity returns to the laser, locking the output of the laser to the frequency of the cavity mode and therefore also increase the coupling efficiency of the radiation into the cavity. When out of resonance, destructive interference occurs within the cavity and no light returns to the laser, so the laser scans normally at its free running wavelength. Phase matching of the fed-back light to the laser is accomplished by a home-written LabVIEW electronic circuit, which provides an error signal through a data acquisition card (DAQ, National Instruments USB-6259, 16 bits resolution, 1.25 MS/s) to control PZT. In perfect phase matching, the periodic tuning of the diode current will result in a symmetrical signal (ratio of V_out_ and V_in_). By differentiating and integrating ratio of V_out_ and V_in_, a signed error signal generated from an asymmetric signal and will be fed to the PZT A until the error signal becomes near zero.

The PZT B is a new design in this sensor system which allows to modulate the length of the cavity. This modulation will change the frequencies of the modes formed in resonance with the cavity and therefore cause a controlled shift in the observed frequency comb, therefore more points across the width of an absorption shape will be recorded. Then, a more precise spectral line fitting and concentrations measurement will be obtained.

The automatic gas distribution system from Environics can provide 12 kinds of mixed gases at different concentrations levels. The standard concentration gas of pure SF_6_ (99.99%) and 50 ppm CO, SO_2_, H_2_S, SO_2_F_2_, HF, CF_4_, CO_2_, COS, and O_2_ (99.99%) are from National Standards Material Center. A valve is placed between the automatic gas distribution system and enhanced cavity to adjust gas flow, while the pressure meter is linked to the enhanced cavity to control the test gas pressure.

## Experimental Method, Results and Discussions

### Cavity locking modes and enhanced performance

**Figure 2:**
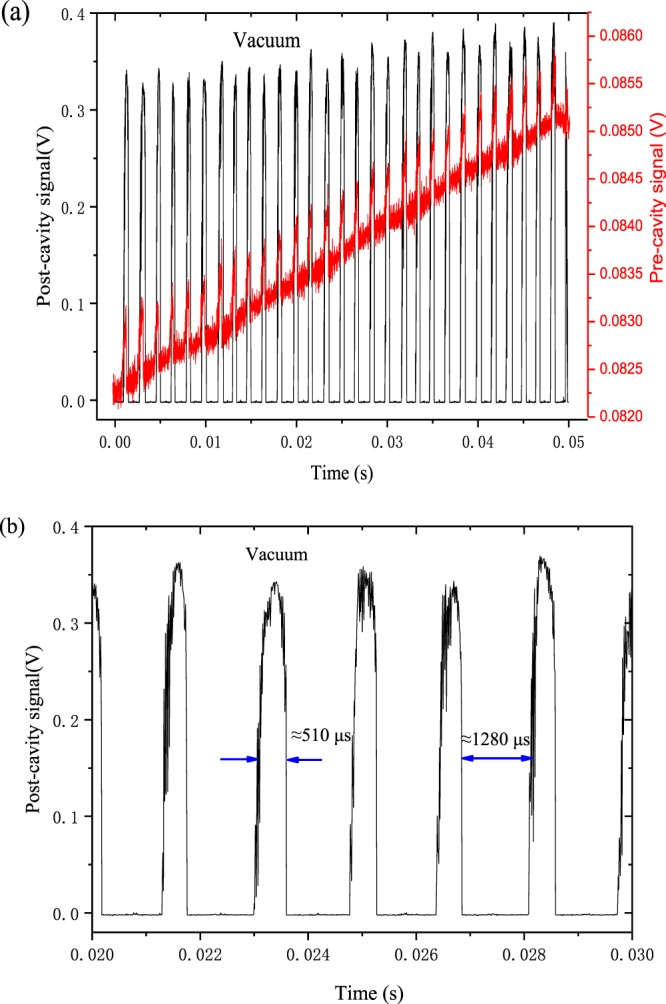
The cavity output signal and post-cavity signal when tuning the applied laser current with a sawtooth signal at 20 Hz. (**a**) Even and odd modes as well as threshold current lowering effect on optical feedback. (**b**) A magnification of the modes between 0.02 s to 0.03 s. Locking time, estimated by the FWHM, is 510 µs. The time between modes is 1250 µs.

By running the home-written LabVIEW electronic circuit of frequency locking with setting the temperature of laser at 31.3 °C and tuning the applied laser current with a sawtooth signal at 20 Hz, the output frequency of laser will scan or lock in the resonance frequency of the enhanced cavity when phase matching happens, as shown in Fig. 2(a) (5000 data points at each scan in 50 ms). As the laser remains locked while the free-running frequency of laser continuously tunes with the applied current, the laser frequency excursion will increase until reaching a maximum. After the maximum point, the laser frequency will scan linearly with applied current until the laser locks to the next available cavity resonant frequency (can be seen from the red line of Fig. 2(a)).

Over 50 ms, the laser locked to 29 successive cavity modes with a locking time of 510 µs estimated by the full width at half maximum (FWHM) of mode, much longer than the ring-down time, *τ*_0_, (typically 10.5 µs, calculated through running cavity ring-down time spectroscopy at the last cavity mode of the applied laser current ramp in vacuum). The time between modes was 1250 µs, which can be seen clearly in Fig. 2(b), which is influenced by the injection efficiency of the laser beam defined as the ratio of the power back to the laser and the output power of the laser. When the injection efficiency is higher, more light is returned to the laser, the locking time is longer.

In Fig. 2(a), there is a clear amplitude oscillation in the cavity output signal for alternating mode separated from each other, called as even and odd modes, because they have a different phase at the folding mirror, and therefore slightly different reflectivity and power loss. The data of the transmitted signal from even and odd modes should be analyzed separately to get a precise absorption of gas. There is also a phenomenon that the output power of laser where modes build up is larger than the power without modes, increased by about 0.0008 V, called as threshold current reduction effect, equal to approximately 1.07 mA, which will help to improve the minimum detection limit of gas sensor.

The effective reflectivity, R, of the sensor is approximately 99.98% at 4285.01 cm^−1^, as determined from *lnR* = *−*(*L*_1_ + *L*_2_)*/*(*2cτ*_0_). For a V-shaped cavity, this reflectivity implies the cavity finesse, F, (=*πR/*(1 − *R*^2^) equal to 7074, which corresponds to 4504 (=2 F/π) passes for the average photon of laser in the cavity. Hence an effective optical path length of 4.5 km is obtained in our V-shaped cavity of only 35 cm length^18^.

### Target Transition Search

50 ppm CO is injected into the cell to 40 torr through a pneumatic valve near the optical input (the central mirror) and can exit through another such valve near the optical output (ends mirror) if necessary. To find the target transition of R(6) at 4285.01 cm^−1^, which is interference free from absorption spectra of SF_6_ mixtures, and to achieve the maximum possible sensitivity with high precision for trace CO in SF_6_ mixtures, a spectral search between 4281.64 cm^−1^ to 4289.86 cm^−1^ is undertaken by adjusting the temperature of laser, tuning the applied laser current and comparing their relative intensity of absorption line with the value from HITRAN database.

### Minimum Detection Limit, Stability, Precision and Linearity

**Figure 3:**
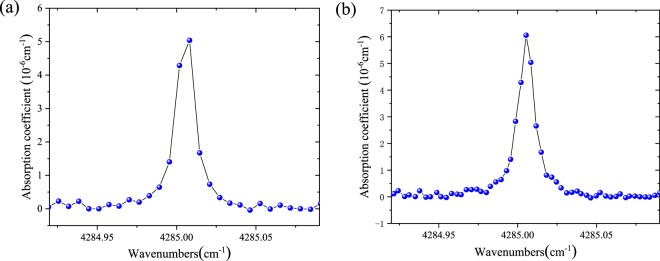
Absorption spectrum of 200 ppb CO in SF_6_ mixtures at 40 torr based on optical feedback V-shaped cavity enhanced absorption spectroscopy measurement. (**a**) Without modes shift. (**b**) With shifting the positions of the modes with approximately half of the free spectral range.

The absorption spectrum of 200 ppb CO, O_2_, SO_2_, H_2_S, SO_2_F_2_, HF, CF_4_, CO_2_, COS in SF_6_ and H_2_O at 40 torr can be obtained as presented in Fig. 3(a). With a fixed cavity mirror, only a few points across the absorption spectral line can be obtained because the modes resolution is only 0.0071 cm^−1^. A low number of points across a molecular absorption feature may lead to difficulties in precisely fitting a line-shape profile, which has consequences for precisely determining the concentration. In Fig. 3(b), by manually adjusting the PZT, the modes have been shifted by approximately half of the FSR, about two times more precise absorption line-shape is obtained. With the help of self-written LabVIEW electronic circuit, the PZT can be automatically controlled to modulate the cavity length as many times as we want. After comprehensive considering of sensor stability and spectral resolution, for this CO sensor, the cavity length has been modulated five times, and the spectral resolution reaches to 0.0015 cm^−1^, and will lead to more precisely line-shape profile fitting.

**Figure 4:**
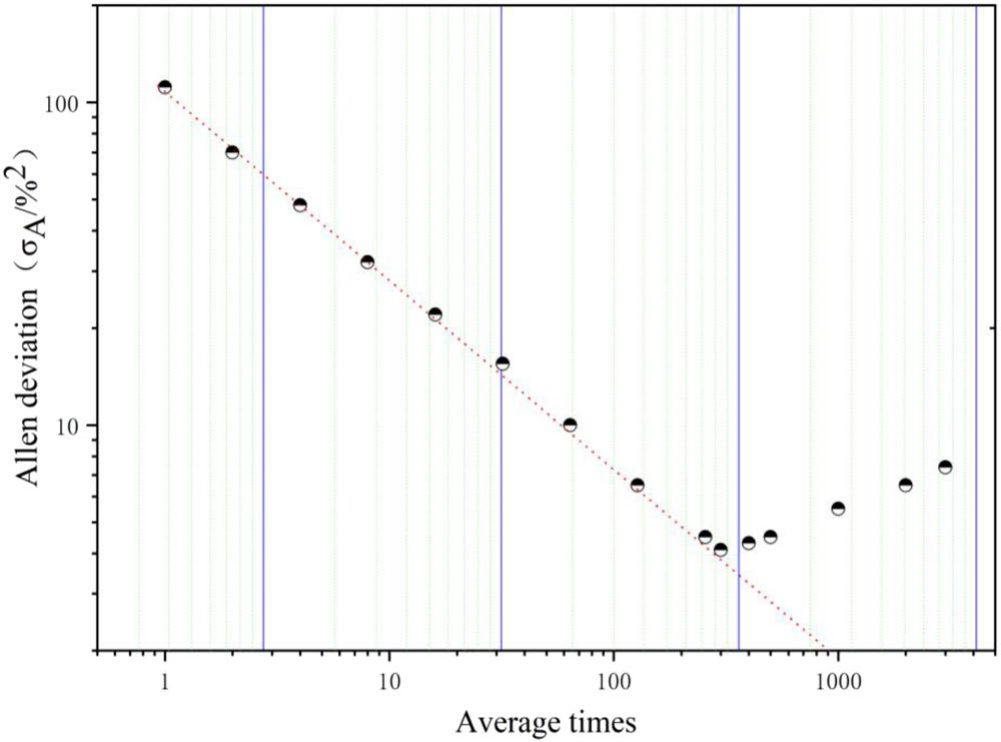
The log-log plot for Allan deviation of CO spectra versus average times. The dashed line indicates white noise behavior, which is dominant until the optimum average times of 120.

To decide the optimum time of integration spectral data, Allan variance analysis^18^ is performed for more than 10,000 consecutive spectra by automatically running LabVIEW electronic circuit. Allan variance log-log plot for this sensor system is shown in Fig. 4, which indicates that average times shorter than about 120, equalling to optimum integration time of 30 s (average times × modulating times of cavity length of 5 × 50 ms), are dominated by a τ^−1/2^ slope, as expected from white noise (dashed line). For more average times, the Allan standard deviation starts to increase as a result of system drifts.

**Figure 5:**
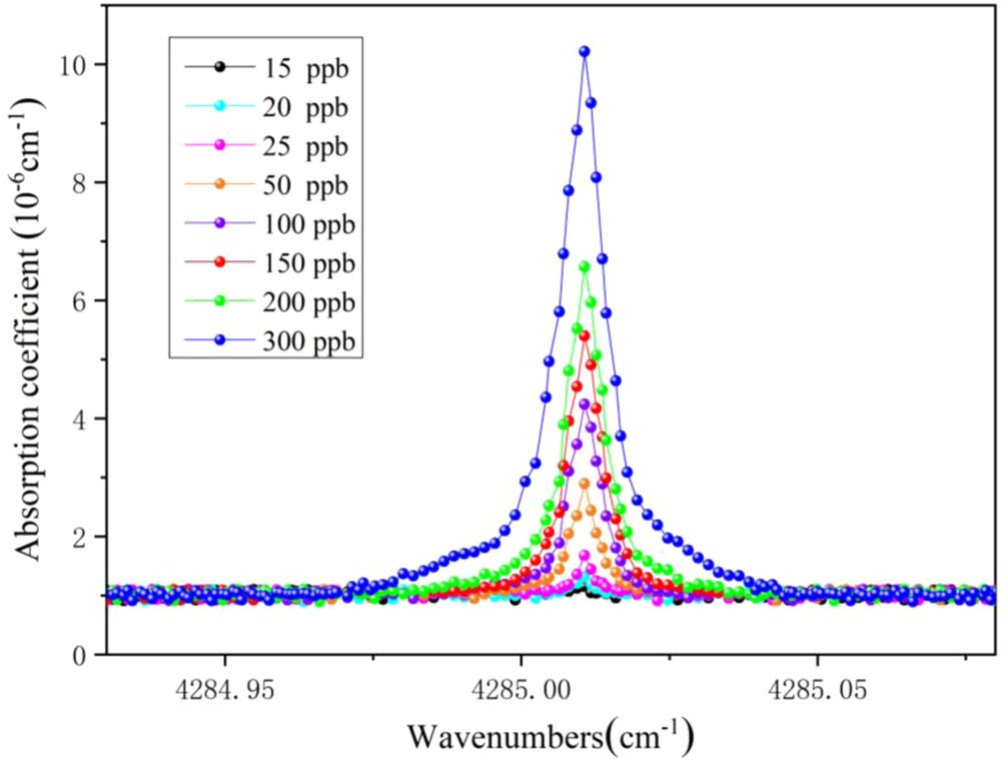
Absorption spectra of CO at different concentrations in SF_6_ mixtures after integration the data of 30 s (40 torr). The spectrum of 20 ppb CO can be clearly seen. The minimum detection limit of CO in SF_6_ mixtures at 40 torr reaches to 18 ppb.

Figure 5 shows absorption spectra of CO at different concentrations in SF_6_ mixtures. The spectrum of 20 ppb CO can be clearly seen. According to the 3*s* rule (*s* is noise standard deviation of absorption spectrum baseline fitting), the minimum detection limit of multi-comb optical-feedback cavity enhanced absorption spectroscopy sensor for CO in SF_6_ mixtures at 40 torr reaches to 18 ppb.

**Figure 6:**
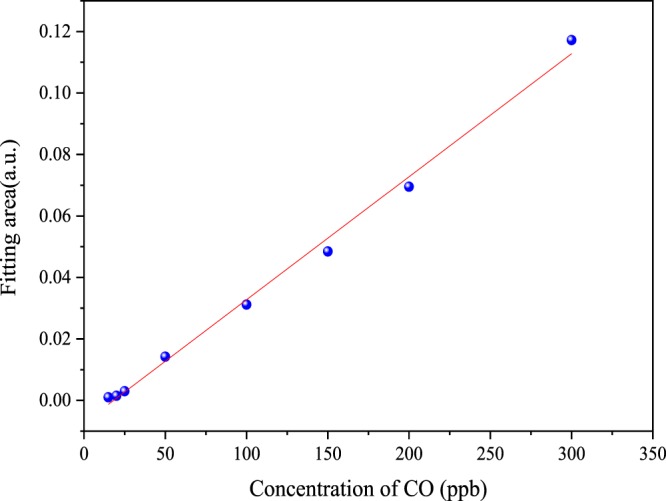
Fitting area of absorption spectra vs CO concentration in SF_6_ mixtures. R-square value is 0.994.

To achieve quantitative analysis of CO samples, a linear relationship between fitting area of absorption of CO and its concentrations is developed based on the least squares method as shown in Fig. 6. The regression equation is as

1 $$FA=4.26271{{\rm{E}}}^{-4}\ast {C}_{CO}-0.00882$$

where *FA* is fitting area of absorption spectra of CO, *C*_CO_ is concentration of CO. R-square value is 0.994, which confirms the excellent linearity of the sensor response to CO concentrations.

**Figure 7:**
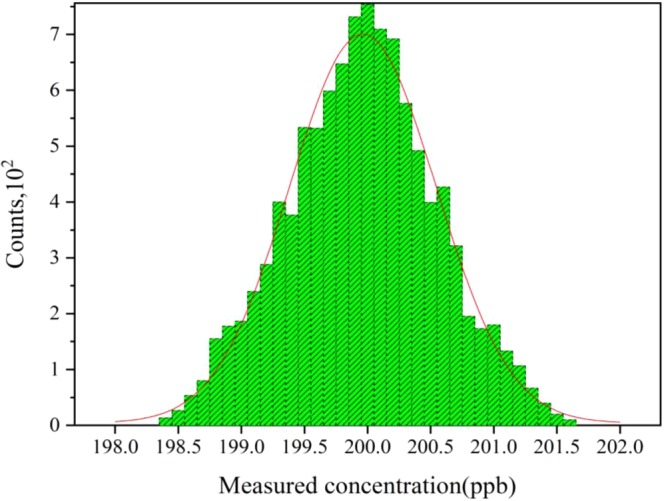
Histogram plot of the deviation the measured concentration compared with the real value of 200 ppb CO. The red line depicts a Gaussian profile, which indicates that the measurement noises are clearly Gaussian distributed The half maximum of the Gaussian profile is 141 ppt with the concentration output rate of 30 s, which verifies that the sensor detection precision is higher better than 150 ppt.

To further examine the stability and precision of this CO sensor system, time series measurements of 200 ppb CO in SF_6_ mixtures are carried out for 5000 mins, which includes 10000 concentration points. In Fig. 7, the histogram plot of measured concentrations of CO show nice Gaussian distributions around the mean value, which indicates that the measurement noises are clearly Gaussian distributed. The FWHM of the Gaussian profile is 141 ppt with the concentration output rate of 30 s, which verifies that the sensor detection precision is higher better than 150 ppt, much better than our undeveloped setup without modes shift of 85 ppb.

**Figure 8:**
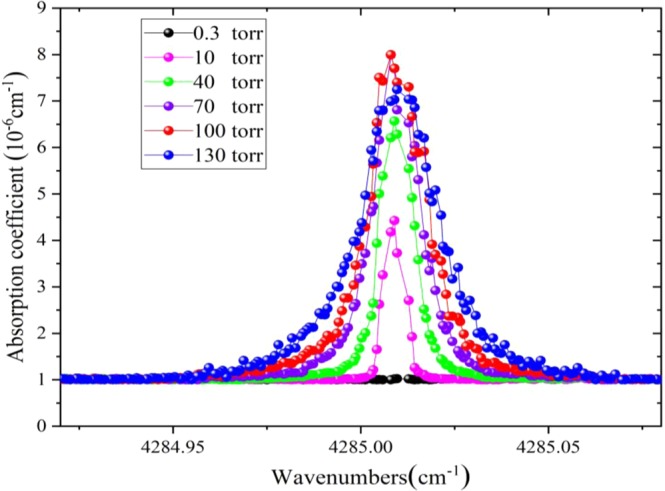
Absorption Spectra of of 200 ppb CO in SF_6_ mixtures at different pressures with optimum integration time of 30 s.

In order to obtain the effect of gas pressure on CO minimum detection limit and detection precision, the absorption spectra of 200 ppb CO in SF_6_ mixtures at different gas pressures are obtained as shown in Fig. 8. Obviously, the peak intensity of absorption spectra at 4285.01 cm^−1^ for CO meets an enhancement along with the increment of gas pressure until 100 torr while FWHM of absorption spectra increases with the increment of gas pressure. The minimum detection limit of CO according to the standard deviation of noise and the detection precision according to time series measurements at different pressures are summarized as in Table 1. The detection limit goes up nonlinearly with the increment of gas pressure, which results from combined influence of number density increase and peak height degradation resulting from pressure broadening. While, the detection precision suffers a small reduce with the enhancement of gas pressure, this is probably because line pressure broadening leads to a worse spectral line fitting.

**Table 1:** Minimum detection limit and detection precision of CO at different gas pressures.

Gas pressure (torr)	Minimum detection limit (ppb)	Detection precision (ppt)
10	55	102
40	18	150
70	13	299
100	9	401
130	5	496

Thus, when measuring gas CO on line, if the minimum detection limit can fulfill operational requirements, there are numerous advantages of working at lower pressure where the line broadening is dominated by the Doppler effect and the width of absorption lines are much narrower. In this case, there is less overlapping of spectral features upon an absorption-free baseline, allowing a more precise spectral line fitting and more precise detection which is very important in industrial measurement. Besides, less gas sample is taken from gas insulated electrical equipment for measuring is better because the key role of SF_6_ is for insulation. The more SF_6_ has been taken for measurement, the worse of insulation ability, the more insulation faults happen.

What is more, the optical sensor can be expanded to detect other SF_6_ decomposition components including SO_2_, H_2_S, SO_2_F_2_, HF, CF_4_, CO_2_ and COS when tuning the same laser through an absorption line of target gas or changing an another DFB laser.

## Conclusions

In this work, we first describe a sensor system based on multi-comb optical-feedback V-shaped cavity enhanced absorption spectroscopy with a near-IR CW diode laser at 2.3 μm for CO detection in SF_6_ mixtures (SF_6_, SO_2_, H_2_S, SO_2_F_2_, HF, CF_4_, CO_2_, COS, O_2_, and H_2_O). Through cavity down time measurement, the effective optical path of 35 cm length V-shaped cavity reached to 4.5 km. A piezoactuator attached to the rear of an end mirror of the V-shaped cavity was constructed to allow the cavity length to be carefully modulated and change the position of the comb of modes. Through modulating the cavity length five times automatically by self-written LabVIEW electronic circuit, the spectral resolution was improved to 0.0015 cm^−1^ from 0.0071 cm^−1^. Based on Allan deviation analysis, optimum integration time was determined as 30 s with spectrum sans of 120 times, the minimum detection limit and detection precision of CO targeting the *R*(6) line at 4285.01 cm^−1^ was better than 18 ppb and 150 ppt (40 torr), respectively. Further experiments at measurement of CO at different gas pressures showed that the minimum detection limit goes up nonlinearly with the increment of gas pressure while detection precision suffers a small reduce with the enhancement of gas pressure. When measuring gas CO in SF_6_ mixtures, the minimum detection limit, detection precision and volume of gas samples should be compromised. In the near future, deployment of this sensor system in gas insulated electrical equipment to further evaluate its performance for on-line monitoring will be conducted.

## References

[1] Brunt RJV, Herron JT (1990). Fundamental processes of SF_6_ decomposition and oxidation in glow and corona discharges. IEEE Trans. Dielectr. Electr. Insul..

[2] Tang J, Liu F, Zhang ZX (2012). Partial discharge recognition through an analysis of SF6 decomposition products part 1: decomposition characteristics of SF6 under four different partial discharges. IEEE Trans. Dielectr. Electr. Insul..

[3] Wang J (2018). High Sensitivity and Selectivity of AsP Sensor in Detecting SF_6_ Decomposition Gases. Sci. Rep..

[4] Zhang XX, Chen QC, Tang J, Hu WH, Zhang JB (2014). Adsorption of SF_6_ decomposed gas on anatase (101) and (001) surfaces with oxygen defect: A density functional theory study. Sci. Rep..

[5] Okabe S, Kaneko S, Minagawa T (2008). Detecting characteristics of SF_6_ decomposed gas sensor for insulation diagnosis on gas insulated switchgears. IEEE Trans. Dielectr. Electr. Insul..

[6] Zhen. D. S. *et al*. Detection method of decomposition products in sulfur hexafuoride electriacal equipment. *China Power Industry standard*, DL/T 1205-2013 (2014).

[7] Lei D, Yu Y, Li C, Tittel FK (2015). Ppb-level formaldehyde detection using a CW room-temperature interband cascade laser and a miniature dense pattern multipass gas cell. Opt. Express..

[8] Liu K (2015). Highly sensitive detection of methane by near-infrared laser absorption spectroscopy using a compact dense-pattern multipass cell. Sensor. Actuat. B-Chem..

[9] Johannes E, Goeran BN, Andreas D, Steven W (2018). Data analysis and uncertainty estimation in supercontinuum laser absorption spectroscopy. Sci. Rep..

[10] Ma. GM (2017). Tracing acetylene dissolved in transformer oil by tunable diode laser absorption spectrum. Sci. Rep..

[11] Werle. P, Mücke. R, Slemr F (1993). The limits of signal averaging in atmospheric trace-gas monitoring by tunable diode-laser absorption spectroscopy (TDLAS). Appl Phys B..

[12] Wang F, Li N, Huang QX, Yan JH, Cen KF (2007). Measurements on co concentration and gas temperature at 1.58 um with tunable diode laser absorption spectroscopy. AIP Conf. Proc..

[13] Chao X, Jeffries JB, Hanson RK (2009). Absorption sensor for CO in combustion gases using 2.3 μm tunable diode lasers. Sci. Meas. Technol..

[14] Dang JM, Yu HY, Sun YJ, Wang YD (2017). A CO trace gas detection system based on continuous wave DFB-QCL. Infrared Phys. Technol..

[15] Ghorbani R, Schmidt FM (2017). ICL-based TDLAS sensor for real-time breath gas analysis of carbon monoxide isotopes. Opt. Express..

[16] Cui Ruyue, Dong Lei, Wu Hongpeng, Li Shangzhi, Zhang Lei, Ma Weiguang, Yin Wangbao, Xiao Liantuan, Jia Suotang, Tittel Frank K. (2018). Highly sensitive and selective CO sensor using a 233 μm diode laser and wavelength modulation spectroscopy. Optics Express.

[17] Wan F, Chen WG, Zhou Q, Zou J (2014). Using a sensitive optical system to analyze gases dissolved in samples extracted from transformer oil. IEEE Electr. Insul. Mag..

[18] Maisons G (2010). Optical-feedback cavity-enhanced absorption spectroscopy with a quantum cascade laser. Opt. Lett..

[19] Hamilton DJ, Orr-Ewing AJ (2011). A quantum cascade laser-based optical feedback cavity-enhanced absorption spectrometer for the simultaneous measurement of CH_4_ and N_2_O in air. Appl. Phys. B..

[20] Chen WG, Wan F, Wang JX (2015). Frequency-locking and threshold current-lowering effects of quantum cascade laser and an application in gas detection field. Chin. Phys. B.

[21] Gorrotxategi-Carbajo P, Fasci E, Ventrillard I (2013). Optical-feedback cavity-enhanced absorption spectroscopy with a quantum-cascade laser yields the lowest formaldehyde detection limit. Appl. Phy. B..

[22] Manfred KM, Ritchie GAD, Lang N, Ropcke J (2015). Optical feedback cavity-enhanced absorption spectroscopy with a 3.24 μm interband cascade laser. Appl. Phys. Lett..

[23] He QX (2018). Dual-feedback mid-infrared cavity-enhanced absorption spectroscopy for H2CO detection using a radio-frequency electrically modulated interband cascade laser. Opt. Express..

